# Bilateral Pseudoarthrosis of the Medial End of the Clavicles: A Rare Case Report with a Two-Year Follow-Up

**DOI:** 10.1155/2020/8847995

**Published:** 2020-10-01

**Authors:** Manuel Bomfim Braga Júnior, Argos Queiroz Alves de Souza, Carlos Augusto Belchior Bitencourt Júnior, Fernando Henrique Uchôa de Alencar, Renan Teixeira Lôbo, Jonatas Brito de Alencar Neto, Réjelos Charles Aguiar Lira

**Affiliations:** ^1^Instituto Doutor José Frota, Serviço de Ortopedia e Traumatologia, Fortaleza-CE, Brazil; ^2^Universidade Federal do Ceará, Faculdade de Medicina, Fortaleza-CE, Brazil

## Abstract

Bilateral clavicle fractures are considered rare. Most of the cases are caused by high energy traumas, such as automobile accidents. Such fracture is related to a higher frequency and severity of associated lesions. In this report, the authors present a twenty-two-year-old male patient's case who, after a motorcycle collision, suffered a bilateral medial end clavicle closed fracture, developing asymptomatic bilateral pseudoarthrosis after the patient refuses surgical treatment. The presented case is quite rare. Aspect related to the patient's evolution during treatment will be discussed, plus the fracture mechanism, associated injuries, the bilateral clavicular fractures treatment, and management in cases of pseudoarthrosis will be analyzed.

## 1. Introduction

Fracture of the clavicle is one of the most common injuries in orthopedic practice. The incidence ranges from 2% to 5% of total fractures in adults [1]. The affected regions, in decreasing order, are middle (80%), lateral (15%), and medial (5%) [2].

However, fractures that affect both clavicles are rare, representing less than 0.5% of all clavicle fractures [1]. In relation to bilateral clavicle fractures, the ratio between men and women is around 2.64 : 1, and the incidence is increasing up to 30 years old, with two peaks of incidence: the first from 21 to 30 years old and the second from 41 to 50 years old [3]. Most bilateral cases involve high-energy trauma, such as automobile accidents [3–8].

We present a patient's case with bilateral fracture of both medial ends of the clavicles evolving with asymptomatic bilateral pseudoarthrosis, after the patient's refusal of surgical treatment. Aspects of trauma mechanisms, associated injuries, type of treatment, and management in cases of pseudoarthrosis will be discussed.

## 2. Case Presentation

Patient, male, 22 years old, blacksmith, victim of motorcycle accident when colliding with a cow.

Clinical examination showed pain on both clavicles and difficulty in moving the shoulders. There were no evidence of skin or muscular lesions. The neurovascular examination of superior members was unchanged. No other associated systemic injuries were found with complete investigation.

**Figure 1:**
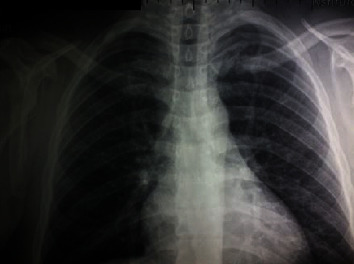
Anteroposterior chest radiography performed at the emergency department, showing fracture of the medial ends of both clavicles.

On the radiographic exam, bilateral fracture of medial ends was evidenced. Both fractures were classified as type III according to the Allman classification, type 1B1 according to the Edinburgh classification, and type 15.1 according to the AO/OTA classification (Figure 1). We opted for conservative treatment, with immobilization in the eight plaster cast.

On the return from the sixth week, anteroposterior radiography of the clavicles was requested, which evidenced the absence of bone callus. Clinically, the patient had no pain and also did not report any complaints regarding the mobility of the upper limbs. When we discussed treatment options with the patient, he refused to undergo surgery, as he had already returned to his activities, still without symptoms. The immobilization in the form of eight was removed, and physical therapy was started.

On the return of the twelfth week, anteroposterior radiography of the clavicles was repeated, showing pseudoarthrosis in both. The patient did not report any difficulty in performing manual labor, and the strength of the limbs was preserved.

**Figure 2:**
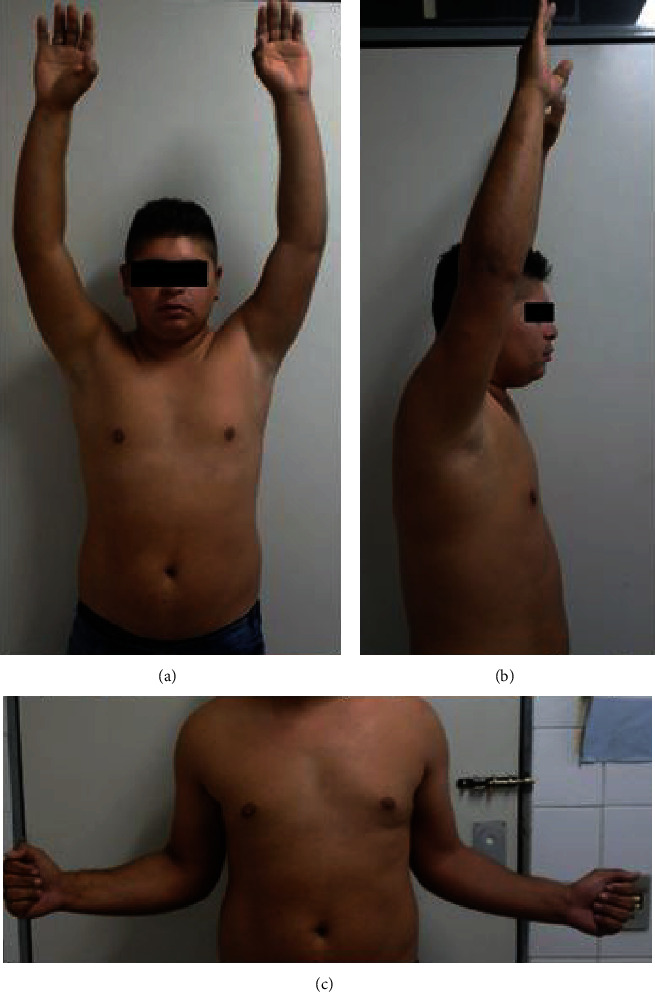
After two years of follow-up, there was complete recovery of shoulder movement. (a) arms elevation—anterior view. (b) arm elevation—side view. (c) external rotation.

**Figure 3:**
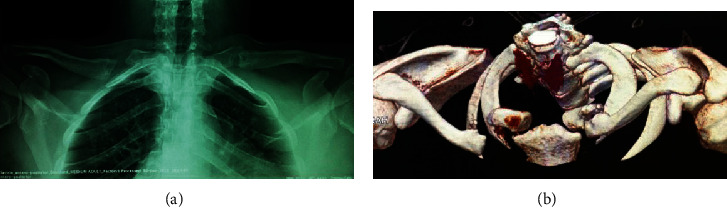
After two years follow up, (a) Anteroposterior radiograph of the clavicles revealing bilateral pseudoarthrosis. Incidence performed with a 40° head rays (serendipity incidence). (b) Computed tomography with three-dimensional reconstruction of the clavicles characterizing the pseudoarthrosis.

On the last return, with two years of follow-up, the patient presented without complaints and even reported that he was able to lift heavy objects on his shoulders. On the physical examination, the patient had complete and painless ROM (range of motion). We applied the DASH questionnaire (Disabilities of the Arm, Shoulder, and Hand), adapted for the Brazilian population [9], whose score was 5.83, showing an excellent functional capacity. There were no changes in active shoulder mobility (Figure 2). We performed a computed tomography with three-dimensional reconstruction in order to better characterize the pseudoarthrosis (Figure 3).

## 3. Discussion

Bilateral fractures of the clavicle are considered rare in orthopedic practice. Throckmorton [10] found 16 cases in a sample of 614 clavicle fractures, with only two cases of bilateral fractures affecting both medial ends.

The mechanisms responsible for causing bilateral fractures include compressive force in the axial direction of the shoulders, direct trauma to both clavicles, and two successive episodes of trauma, one on each shoulder [6–8].

Associated systemic injuries are more common than in unilateral fractures and tend to be more severe, usually inserted in the context of polytrauma [3]. Associated injuries reported include severe chest injuries [5, 6, 8](multiple rib fractures, unstable chest, hemothorax, and hemopneumothorax); skull injuries (fractures of parietal and occipital bones, subdural hematoma, and pneumoencephalic); scalp laceration or avulsion [8]; brachial plexus injury [5]; scapular fracture; pelvis fracture [4]; lower limb fractures; and spleen rupture [6]. Unlike the two patients with bilateral fracture of the medial ends mentioned in the epidemiological study by Throckmorton [10], who died from associated injuries, and the patient reported by Brunner [6], with multiple injuries to the lower limbs and spleen rupture, no associated injuries were found in the patient in this report.

The treatment for bilateral fractures varies widely in the literature. Among the methods used are conservative treatment [3–5, 7], fixation with plates [3, 6], and external fixation [4]. In a literature review, Van den Bout [3] states that the open reduction and internal fixation should be the mainstay of treatment for bilateral fractures of the clavicle.

Bonnevialle [4] reports a case of failure in conservative treatment, obtaining bone consolidation when he changed to surgical treatment with external fixators. The author justified the use of external fixators because of a suspected infection at the fracture site.

Among the reasons for indicating surgical treatment, the following stand out: the deviation between the fragments and the decrease in the patient's inspiratory capacity [3]. Van den Bout [3] recorded bone healing and recovery of shoulder mobility after treatment with internal fixation. The author considered internal fixation necessary due to fracture deviations.

We found in the literature only one case report, described by Brunner [6], specifically about bilateral fractures in medial ends of traumatic origin. After the previous unsuccessful conservative treatment of clavicle fractures, it was decided to undergo surgical treatment with blocked angled plates. Even with the breaking of one of the plates, there was a recovery of range of motion and improvement of pain, six weeks after surgery.

With regard to bilateral clavicle fractures, pseudoarthrosis is more common after conservative treatment, compared to surgical treatment [3]. In the case of a mildly symptomatic patient, Hargan [7] did not require surgical treatment for pseudoarthrosis, whereas Mullet [5] improved the patient's pain after surgical treatment of the pseudoarthrosis, with internal fixation and bone graft.

Upon the return of our patient, six weeks after the trauma, the radiological examination continued to demonstrate the absence of consolidation of the bone fragments, characterizing a delay in the consolidation. However, the patient had no pain or limited movement. We discussed treatment options with the patient, who chose not to undergo surgery, since he had already returned to his usual activities without any symptoms. Pseudoarthrosis was then conducted conservatively, evolving well with good functional capacity and without limitations, with good functional score at two years' follow-up.
